# Acute Mesenteric Ischemia: A Challenge for the Acute Care Surgeon

**DOI:** 10.1177/14574969211007590

**Published:** 2021-04-19

**Authors:** J. M. Kärkkäinen

**Affiliations:** Heart Center, Kuopio University Hospital, Kuopio, Finland

**Keywords:** Acute Mesenteric Ischemia, Chronic Mesenteric Ischemia, Acute intestinal ischemia, Chronic intestinal ischemia, Endovasular

## Abstract

Acute mesenteric ischemia is considered uncommon, but it appears to be more frequent cause of acute abdomen than appendicitis or ruptured abdominal aortic aneurysm in elderly patients. Surgical treatment without revascularization is associated with high overall mortality, up to 80%. The modern treatment of acute mesenteric ischemia requires collaboration of gastrointestinal surgeons, vascular surgeons, and interventional radiologists. Early revascularization may reduce the overall mortality associated with acute mesenteric ischemia by up to 50%. Clinical suspicion and contrast-enhanced computed tomography performed at early stage are keys to improve outcomes of acute mesenteric ischemia treatment. This review summarizes what the acute care surgeon needs to know about acute mesenteric ischemia with special emphasis on slowly progressing “acute on chronic” mesenteric ischemia.

## Introduction—From Past to Present

Back in the 1990s, acute mesenteric ischemia (AMI) had a dismal prognosis. This is very well demonstrated in a study by Järvinen et al. (1), which included 214 consecutive patients treated for arterial AMI in a single Finnish academic institution between the years 1972 and 1990. At the time, one-third of the patients were treated with bowel resection without revascularization; this was associated with 50% mortality. Two-thirds received only palliative care, typically after an exploratory laparotomy, and the mortality of these patients was 100%. Revascularization was attempted rarely, only in 7% of the cases, and the mortality associated with surgical revascularization of the mesenteric arteries was a discouraging 87%. The overall 30-day mortality of all 214 patients with a median age of 75 years was 82%. The depressing results of AMI treatment lead to decades of diagnostic and therapeutic nihilism, which still continues in many places due to lack of awareness or resources for emergency vascular interventions. Numerous publications dealing with AMI begin with an off-putting phrase declaring AMI as a rare cause of acute abdominal pain with dauntingly high mortality.

Twenty years after the publication by Järvinen et al., we repeated a similar survey in Kuopio University Hospital, an academic institution in Eastern Finland. At the time, computed tomography (CT) was quickly becoming a standard investigation for acute abdominal pain and was being performed with high volume in the emergency department. Prior to the study, we had started training our emergency department radiologists to spot the signs of AMI and report the patency of the mesenteric arteries routinely, rather than sporadically, in the CT examinations in patients with acute abdominal pain. Moreover, we had been using endovascular revascularization in patients with AMI for several years with increasing success rate. During a 5-year study period between 2009 and 2013, 66 consecutive patients with arterial AMI were treated in our hospital (2). Of those, 50 patients (76%) were treated initially with endovascular revascularization; whereas, 16 patients (24%) were primarily managed with bowel resection or mere palliative care with or without explorative laparotomy. The technical success rate of endovascular revascularization was 88%, and 34% required concomitant bowel resection. Three out of six patients with failed endovascular treatment attempt were treated with subsequent surgical revascularization. The 30-day mortality of those 50 patients who received an initial attempt at endovascular revascularization was 32%. This was a good outcome considering that the mean age of these patients was 79 years. The overall mortality of all 66 consecutive patients with AMI treated in our hospital during the study period was 42%.

Although these two Finnish cohorts (1, 2) of different time periods are not directly comparable, the aggressive “endovascular first” strategy had certainly paid off and resulted in roughly 40% absolute reduction in overall mortality associated with AMI. What we also saw was a remarkable change in the attitudes of emergency room physicians and radiologists, vascular surgeons, gastrointestinal surgeons, and interventional radiologists toward the active treatment of these patients. The diagnosis of AMI was no longer seen as a death sentence. More importantly, AMI awareness among physicians working in the emergency department lead to more and faster diagnoses. Today, we no longer consider AMI as a rare entity among the elderly patients with acute abdomen in our emergency unit.

## Definition of AMI

**Fig. 1.**
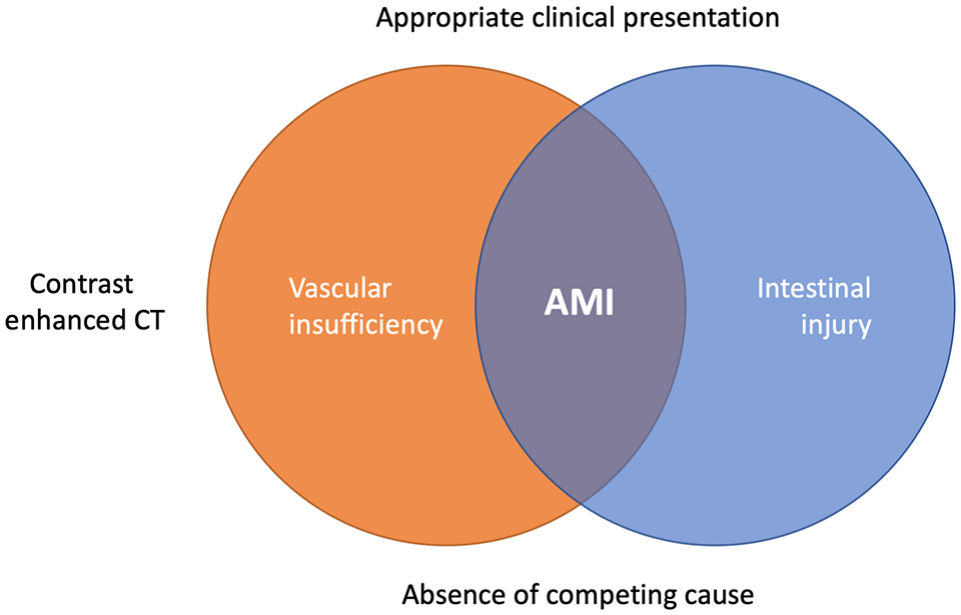
The diagnosis of acute mesenteric ischemia (AMI) is based on findings of vascular insufficiency and signs of intestinal injury in contrast-enhanced computed tomography (CT) accompanied with appropriate clinical presentation.

There is no clear-cut definition for AMI in the literature. The cornerstones of the diagnosis are contrast-enhanced CT and clinical suspicion. AMI is characterized by the presence of mesenteric vascular insufficiency and intestinal ischemic injury in the CT, accompanied with appropriate clinical symptoms in the absence of a competing cause (Fig. 1) (3). The CT signs of intestinal injury in the early phase of AMI may be subtle and no single laboratory exam can rule out AMI; this is especially true for slowly progressing “acute on chronic” mesenteric ischemia. Early diagnosis is vital for successful treatment. For treatment planning, it is also important to distinguish between reversible bowel ischemia and non-reversible transmural bowel necrosis.

The European Society for Vascular Surgery (ESVS) defines AMI as the occurrence of an abrupt cessation of the mesenteric blood flow with development of symptoms that may vary in time of onset from minutes (in embolism) to hours (in atherothrombosis) (4). United European Gastroenterology guidelines for chronic mesenteric ischemia describe the symptoms of chronic mesenteric ischemia as frequent abdominal pain with postprandial worsening, starting 10–30 min after a meal and lasting 1–2 h (5). To avoid postprandial pain, 90% of patients adapt their eating pattern and weight loss is common. In the ESVS guidelines, “Acute on chronic” mesenteric ischemia is defined as AMI in patients with whom symptoms of chronic mesenteric ischemia worsened over the preceding weeks with periods of prolonged and more severe pain, pain without eating, onset of diarrhea, or inability to eat at all.

## Etiological Categorization of AMI

**Fig. 2.**
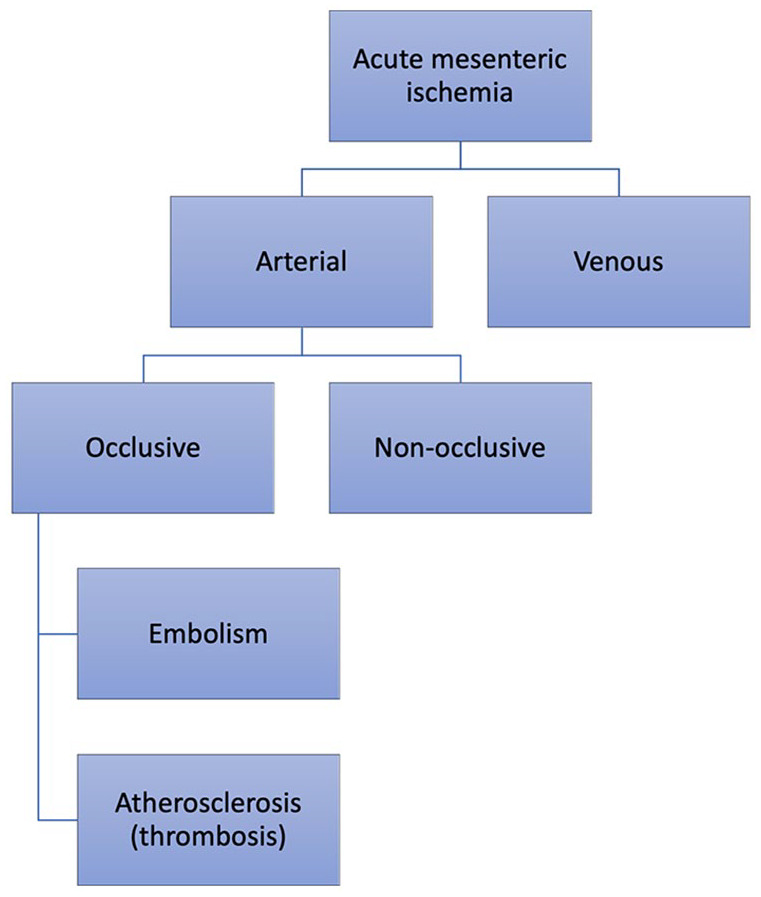
The etiological categorization of acute mesenteric ischemia.

There are four different etiological categories of AMI that need to be distinguished as they differ in treatment and prognosis. These are superior mesenteric artery (SMA) embolism, atherosclerotic SMA occlusion (thrombosis), non-occlusive mesenteric ischemia (NOMI), and venous mesenteric ischemia (Fig. 2). SMA embolism and thrombosis are often referred to as arterial AMI or occlusive AMI. The treatment of arterial occlusive AMI, consisting of embolic and atherosclerotic (thrombotic) etiologies, aims at rapid restoration of blood flow to the SMA by open or endovascular revascularization. The term “occlusive” may be misleading, since even a tight SMA stenosis without a total occlusion may be hemodynamically significant enough to cause AMI in the presence of a diseased celiac artery and inferior mesenteric artery. NOMI refers to AMI without any hemodynamically significant obstruction in the mesenteric arteries that is usually caused by another underlying acute condition causing low cardiac output or vasoconstriction of the mesenteric arteries. Venous mesenteric ischemia is typically caused by mesenteric venous thrombosis. There is another review on mesenteric venous thrombosis in this special issue of Scandinavian Journal of Surgery (6).

## Incidence of AMI

**Fig. 3.**
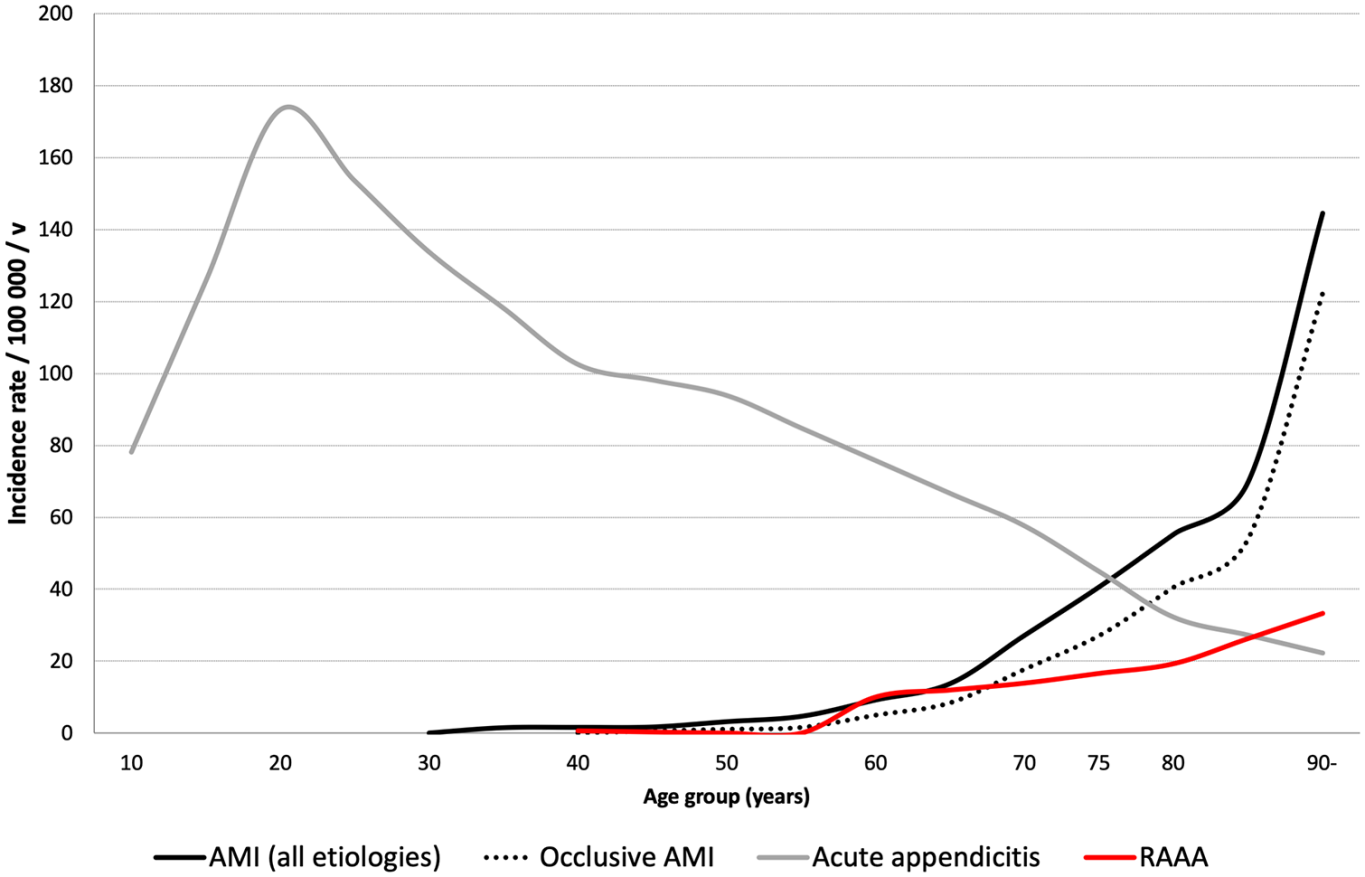
The age-specific incidence rates of acute mesenteric ischemia, acute appendicitis, and ruptured abdominal aortic aneurysm (RAAA) in patients treated in Kuopio University Hospital, Finland, between years 2009 and 2013.

In two contemporary Swedish series of AMI, the reported incidence rates of arterial occlusive AMI were between 5.3 and 5.4 per 100,000 per year (7, 8). From the year 2009 to 2013 in Kuopio, Finland, the incidence rate of AMI was 7.3/100,000/year for all etiologies and 4.5/100,000/year for arterial occlusive AMI (9). These incidence rates may seem low, but it is important to recognize that the incidence of AMI increases exponentially with age. In elderly patients, AMI is a more prevalent cause of acute abdominal pain than, for example, ruptured abdominal aortic aneurysm or appendicitis (Fig. 3) (9). Hence, the diagnostic spectrum of acute abdomen is very different in a 50-year-old patient compared to an 80-year-old patient.

## Clinical Presentation of Embolic AMI

The symptom onset of embolic AMI may be sudden because of an abrupt occlusion of a previously patent SMA. However, the clinical presentation varies depending on the location of the embolic clot within the mesenteric arterial tree. The most severe bowel ischemia is caused by a total occlusion in the root of the SMA, whereas a more distal occlusion typically leaves part of the bowel perfused. The treatment aims at rapid restoration of blood flow to the mesentery, which can be achieved with open embolectomy or endovascular mechanical aspiration embolectomy. A very distal embolic clot in the jejunal or ileocolic side branches of the SMA may be undetectable in the CT and appears as a short segmental necrosis of the jejunum or ileum in the laparotomy; a simple bowel resection with subsequent anticoagulation therapy may be good enough as a treatment in these rare cases. Concomitant embolism in other solid organs (such as the spleen, liver, kidneys, or even the brain) is common in patients with SMA embolism (2).

The common risk factors for embolic AMI are atrial fibrillation, congestive heart failure, prior embolic events (such as a stroke), and recent myocardial infarction, which may be associated with a cardiac thrombus (10). In our series of 18 patients with embolic AMI, 60% had acute onset with symptom duration less than 24 h, 72% had atrial fibrillation, and only one-third were on anticoagulation therapy prior to hospitalization; C-reactive protein (CRP) ranged from normal to more than 200 mg/L and arterial lactate was elevated in less than half of the patients (2). Thus, CRP and lactate may be normal in the early phase of AMI, and therefore, cannot be used to rule out the diagnosis. Of note, troponin T was elevated in half of the patients with embolic AMI. It should be kept in mind that there could be a silent on-going cardiac infarction behind any acute embolic event. It has been shown that 20% to 60% of patients with AMI are admitted to non-surgical emergency room such as internal medicine, and this may cause a significant delay of the diagnosis (2, 11). In addition, D-dimer may be elevated in embolic occlusion of the SMA (4). The ESVS guidelines recommend D-dimer as an exclusion test for AMI. Although this may be true in cases of embolic AMI and fulminant AMI with bowel necrosis, we do not use it to screen patients with acute abdomen in our institution because of poor specificity of the test and uncertainty especially in cases of acute-on-chronic mesenteric ischemia.

## Clinical Presentation of Atherosclerotic (Thrombotic) AMI

The clinical presentation of AMI caused by atherosclerotic occlusive disease is often more obscure and the symptoms can develop either suddenly or over several days depending on the extent and acuteness of the arterial occlusion. A sudden onset of acute abdominal pain may develop when a previously patent SMA occludes suddenly due to acute thrombosis of an underlying atherosclerotic lesion. However, if the SMA was already chronically severely obstructed with sufficiently developed collaterals, an acute thrombosis of the SMA may not essentially change the total blood flow to the mesentery. Moreover, quite often, AMI develops upon severely obstructed or chronically occluded mesenteric arteries, typically involving all three mesenteric arteries, without any evidence of acute thrombosis. This is the classic presentation of “acute on chronic” mesenteric ischemia. In a series of 37 patients with AMI caused by atherosclerotic occlusive disease, less than half presented with clearly visible thrombotic clot in contrast-enhanced CT while the other half presented with chronic calcified obstruction of the SMA and other mesenteric arteries; more than 90% of the patients with atherosclerotic AMI had two or three diseased mesenteric arteries involving both celiac artery and the SMA (12). Hence, the atherosclerotic SMA obstruction in AMI can be either acute or chronic, thrombotic or calcified; these are all manifestations of the atherosclerotic vascular disease.

The risk factors for atherosclerotic AMI are essentially the same as for coronary artery disease and peripheral artery disease. In addition, prolonged hypotension, hypovolemia, and hypercoagulable states have been associated with the risk of atherosclerotic AMI (13).

In our patients with AMI caused by atherosclerotic occlusion or obstruction of the mesenteric arteries, almost half had symptoms for more than 3 days before the diagnosis was made (2). Less than 20% presented with acute onset, defined as less than 24 h duration of symptoms (12). All patients had elevated CRP typically above 100 mg/L and white blood cell count above the normal values ranging from 11 to 19 × 10^9^/L. Arterial lactate was elevated in half of the patients. While abdominal pain was the predominant symptom in 94%, half presented with either concomitant diarrhea or vomiting, or both. Up to 25% had a recorded history of symptoms consistent with chronic mesenteric ischemia and half had a prior diagnosis of coronary artery disease and also half had peripheral artery disease.

## Acute-on-Chronic Mesenteric Ischemia

Prior history of symptoms of chronic mesenteric ischemia (postprandial abdominal pain, fear of eating, and weight loss) is common in the acute-on-chronic presentation pattern of AMI. These premonitory symptoms have been reported prior to the final admission in 25%–84% of patients with AMI caused by atherosclerotic mesenteric vascular disease (2, 14). In a Swedish study of 55 patients referred for endovascular treatment of symptomatic mesenteric ischemia (acute or chronic), it was noted that previous hospitalizations for the same complaints had occurred in 78% who were initially treated with medical therapy alone (14). This highlights the difficulty of the diagnosis, or maybe, the negligence of the findings of vascular insufficiency in the CT examination. Some patients had even undergone exploratory laparotomy or cholecystectomy for gastrointestinal issues without relief of symptoms before the disease culminated in AMI. Although many experimental laboratory tests have been investigated as possible diagnostic markers for AMI, these have not been evaluated in the setting of acute-on-chronic mesenteric ischemia (15). Unfortunately, there are currently no blood tests that could be used widely in patients with acute abdominal pain to screen for AMI in a way similar to the troponin test that is used for screening acute myocardial infarction in patients with acute chest pain (16).

The diagnosis and the presentation pattern of atherosclerotic AMI is much more complicated than that of embolic AMI (16). There are often other factors besides the obstruction of the mesenteric arteries that could contribute to the development of acute bowel ischemia. These include anemia, dehydration, low cardiac output, or major surgery. Sometimes, mere fluid resuscitation, correction of anemia, and administration of antibiotics may reverse the acute intestinal ischemia. However, the patient will remain at risk of recurrent acute-on-chronic episode unless treated with revascularization. Interestingly, even though many patients with atherosclerotic AMI in our institution had several days of symptoms prior to the hospitalization, the prognosis of these patients after revascularization was favorable in comparison to those with more acute onset (2). Thus, the time-window for treatment is extremely variable. The old notion that irreversible bowel damage occurs within 6 h after complete vascular occlusion has little significance in the clinical decision-making (17).

## NOMI

In systemic circulatory failure, the blood flow is redistributed to vital organs, and consequently, vasoconstriction of the mesenteric arteries may cause severe intestinal hypoperfusion despite the mesenteric arteries being patent. This is the definition of NOMI. The typical clinical scenarios in which NOMI may develop are heart failure, hypotension, hypovolemia, sepsis, and abdominal compartment syndrome (18). The use of vasoconstrictive medication (inotropes) or intra-aortic balloon pump may increase the risk of NOMI. Other risk factors include hypotension caused by dialysis or major surgery, especially cardiac or aortic surgery. The diagnosis of NOMI is challenging, because the patients are often intubated and sedated in the intensive care unit. The initial treatment is conservative and aims at restoring intestinal perfusion by treating the underlying condition. Worsening metabolic acidosis and distended abdomen may indicate bowel necrosis and are indications for laparotomy. CT is performed to rule out occlusive AMI and to detect signs of bowel gangrene such as dilated poorly enhancing thin-walled colon. Endovascular stenting is an option to consider whenever a hemodynamically significant SMA stenosis complicates NOMI.

Infusion of vasodilator drugs directly into the SMA via endovascularly placed catheter has been used to treat the mesenteric vasospasm in NOMI. Papaverine, prostaglandin, and iloprost have been used for the purpose. For example, papaverine can be administrated as a 60 mg bolus followed by infusion at the rate of 30–60 mg/h (19). Recently, a systematic review of 12 retrospective studies of interventional local vasodilatory treatment in NOMI reported 40% overall mortality and low treatment-related complication rate of less than 3%. In four comparative studies between patients receiving intra-arterial vasodilator therapy and those who received standard care, the pooled odds of death was significantly lower for those receiving the experimental therapy (20). Another interesting experimental treatment that may be worth studying further is the administration of prostaglandin or iloprost intravenously to improve the hepato-splanchnic blood flow and oxygen intake in NOMI (21, 22). There are no randomized studies and the evidence for these experimental treatments are low; but, in the light of the evidence, there is no wrongdoing in attempting the vasodilator therapy in well-selected patients with NOMI.

## Ct Signs of AMI

**Fig. 4.**
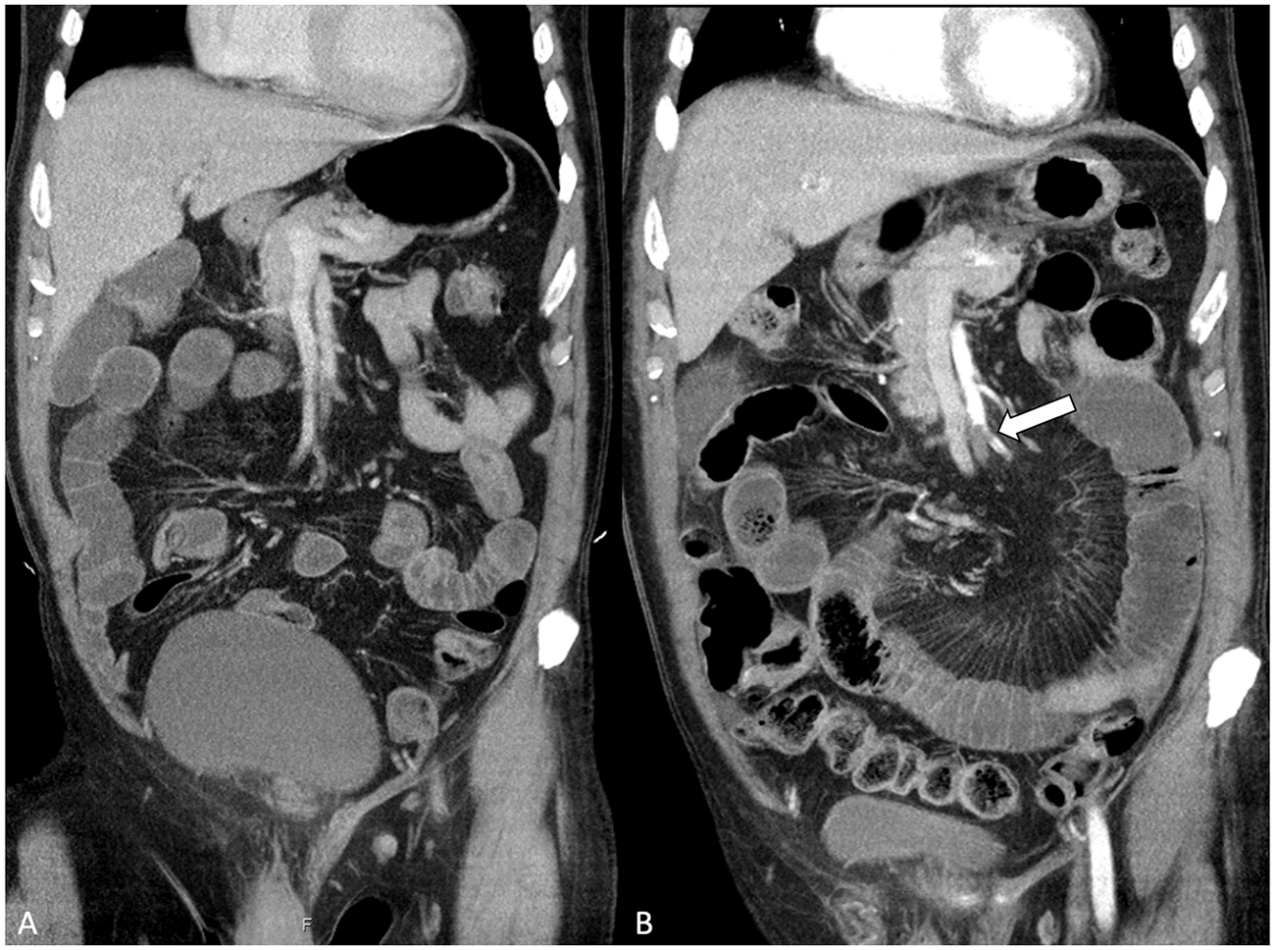
A) The contrast-enhanced computed tomography (CT) of acute abdomen performed in venous phase alone without suspicion of acute mesenteric ischemia (AMI) prior to the imaging. The embolic occlusion of the superior mesenteric artery (SMA) is vaguely visible and was missed in the initial CT examination. B) A second imaging was obtained, this time with suspicion of AMI mentioned in the CT referral. The second CT was performed with split bolus protocol, which consists of contrast enhancement in venous phase and a second bolus in the arterial phase. This time, the SMA embolus is clearly visible (arrow).

Contrast-enhanced CT is the key to diagnosing AMI, and at present, CT is performed with a low threshold for elderly patients with acute abdomen. When AMI is suspected, CT should be performed with contrast enhancement in arterial and venous phases (biphasic or split bolus protocol) (23). The arterial phase enables accurate detection of vascular insufficiency and the venous phase is required for the assessment of bowel wall and solid organ perfusion, and other pathology. The sensitivity and specificity of biphasic CT in AMI has been estimated as 89%–100% in two systematic reviews (24, 25). However, this should be interpreted with caution and the accuracy of CT may be overestimated in AMI. The individual studies in the two reviews were performed exclusively in patients with clinical suspicion of AMI prior to the imaging, and roughly 70%–100% of the study patients had advanced bowel ischemia. In practice, however, AMI is rarely suspected prior to imaging and without clinical suspicion, the CT of the acute abdomen is typically performed in venous phase alone. This may lead to misdiagnosis and delay the treatment (Fig. 4). More importantly, AMI should be diagnosed early when the definitive signs of bowel ischemia may still be absent and the diagnosis is based on clinical suspicion, findings of vascular insufficiency, and unspecific CT findings of possible intestinal injury (Table 1) (26, 27).

**Table 1:** Computed tomography findings in acute mesenteric ischemia.

Vascular findings	• Arterial embolus (oval-shaped clot in a previously unaffected artery) • Arterial thrombus (clot with superimposed calcified lesion) • Mesenteric atherosclerosis • Mesenteric venous thrombosis • Portomesenteric venous gas
Intestinal findings	• Abnormal bowel wall enhancement (decreased, increased) • Bowel wall thickening (edema, hyperdense hemorrhage) • Luminal dilatation (paralysis) • Pneumatosis intestinalis
Other intra-abdominal findings	• Mesenteric fat stranding (edema) • Ascites • Free gas • Solid organ infarction

We performed a retrospective review of 95 consecutive patients treated for AMI in our institution between 2009 and 2013 (12). Clinical suspicion was mentioned in the CT referral in only 31% of the cases prior to imaging. The crucial findings of AMI had been correctly identified in 97% of the radiology reports if the clinician had mentioned suspicion of AMI in the referral; whereas, the corresponding rate was significantly lower, 81%, in cases without clinical suspicion. Patients without clinical suspicion of AMI prior to imaging were more prone to undergo bowel resection. In general, intestinal findings were more difficult to detect than the vascular findings. In retrospect, vascular insufficiency was detectable in 92% of cases with embolic AMI and 100% in atherosclerotic AMI, and at least some evidence of intestinal abnormality attributable to ischemic injury was found in 92% of cases with embolic AMI, 100% in atherosclerotic AMI, and 100% in NOMI.

**Fig. 5.**
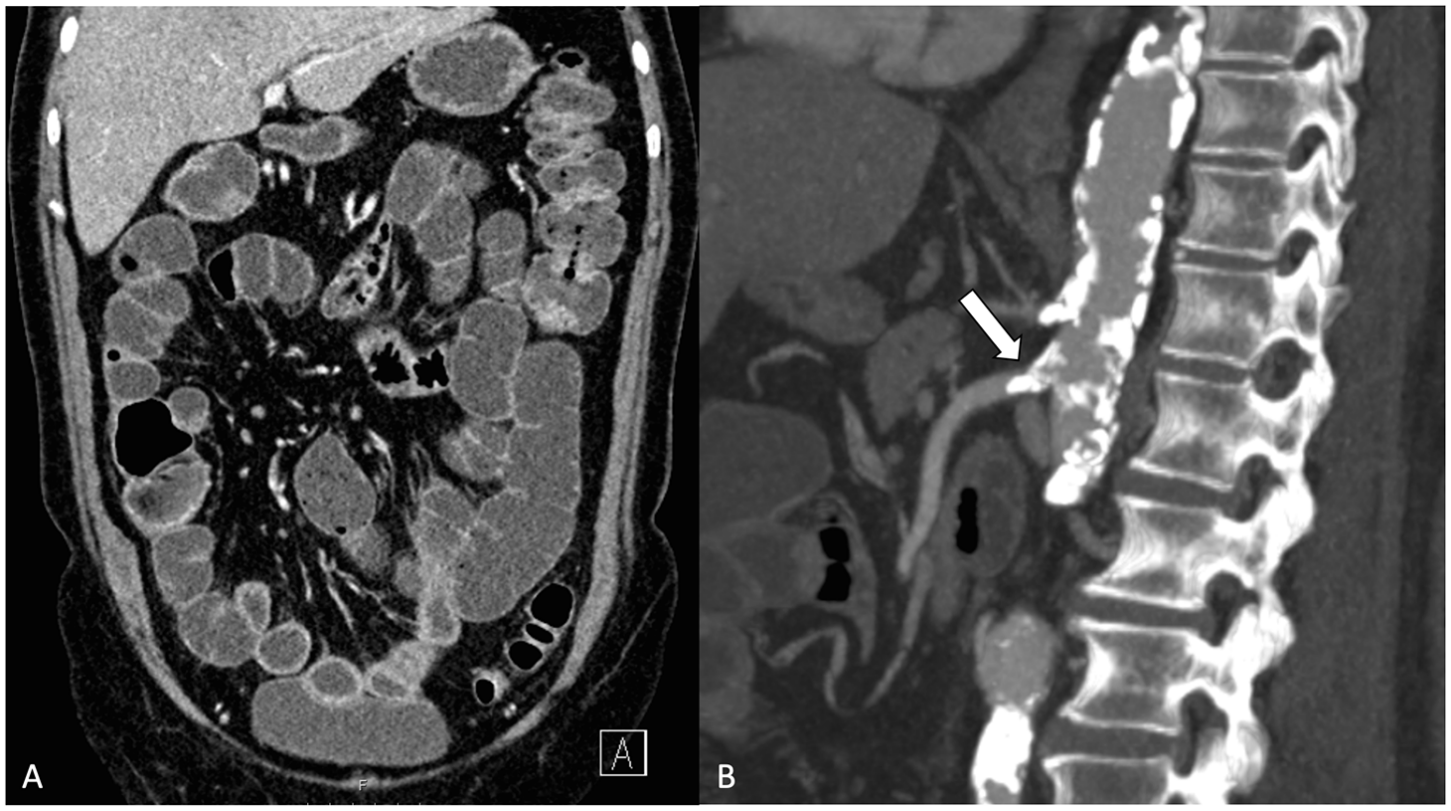
This 70-year-old patient had suffered from postprandial pain and diarrhea for 2 months prior to the final admission. The symptoms worsened and became persistent, and the patient was admitted to the emergency room. C-reactive protein was 400 mg/L. A) Computed tomography showed dilated small bowel loops, bowel wall thickening, mesenteric fat stranding, and abnormal increased enhancement of the intestine. B) All mesenteric arteries were severely obstructed by atherosclerotic disease. Based on the vascular and intestinal findings in the imaging, appropriate clinical presentation, and absence of a competing cause, the diagnosis of acute-on-chronic mesenteric ischemia was made. An exploratory laparotomy was performed mainly due to the high inflammatory markers and clinical suspicion of bowel necrosis. The small bowel appeared pale and cyanotic. However, there was no irreversible bowel damage, and subsequent endovascular stenting of the proximal superior mesenteric artery stenosis (arrow) was performed. The patient recovered without need for bowel resection.

In another study, we compared CT findings of 27 patients with atherosclerotic AMI with 20 patients with intermittent chronic mesenteric ischemia (28). All images were evaluated by three experienced radiologists who knew that the patients were suspected of mesenteric ischemia but blinded to whether the case was acute or chronic mesenteric ischemia. One-third of the patients with atherosclerotic AMI presented without any ischemia-specific CT signs (defined as thrombotic SMA clot, absent or decreased bowel wall enhancement, or pneumatosis). The presence of ischemia-specific CT signs was noted in 77% of patients who required bowel resection after endovascular revascularization, and in 50% of patients who did not need bowel resection. Thus, pneumatosis and decreased bowel wall enhancement are suggestive but not definitive signs of irreversible bowel necrosis (12, 27). Furthermore, the study showed that all patients with AMI had at least some level of abnormal intestinal findings in their CT scans when examined carefully, such as mesenteric fat stranding in 96%, bowel lumen dilatation in 93%, and bowel wall thickening in 70% (Fig. 5). These should be considered as possible signs of early ischemic injury. Only few of the control group patients with intermittent chronic mesenteric ischemia had such findings in their CT scans due to chronic ischemic colitis.

## Treatment Algorithm of Arterial AMI

**Fig. 6.**
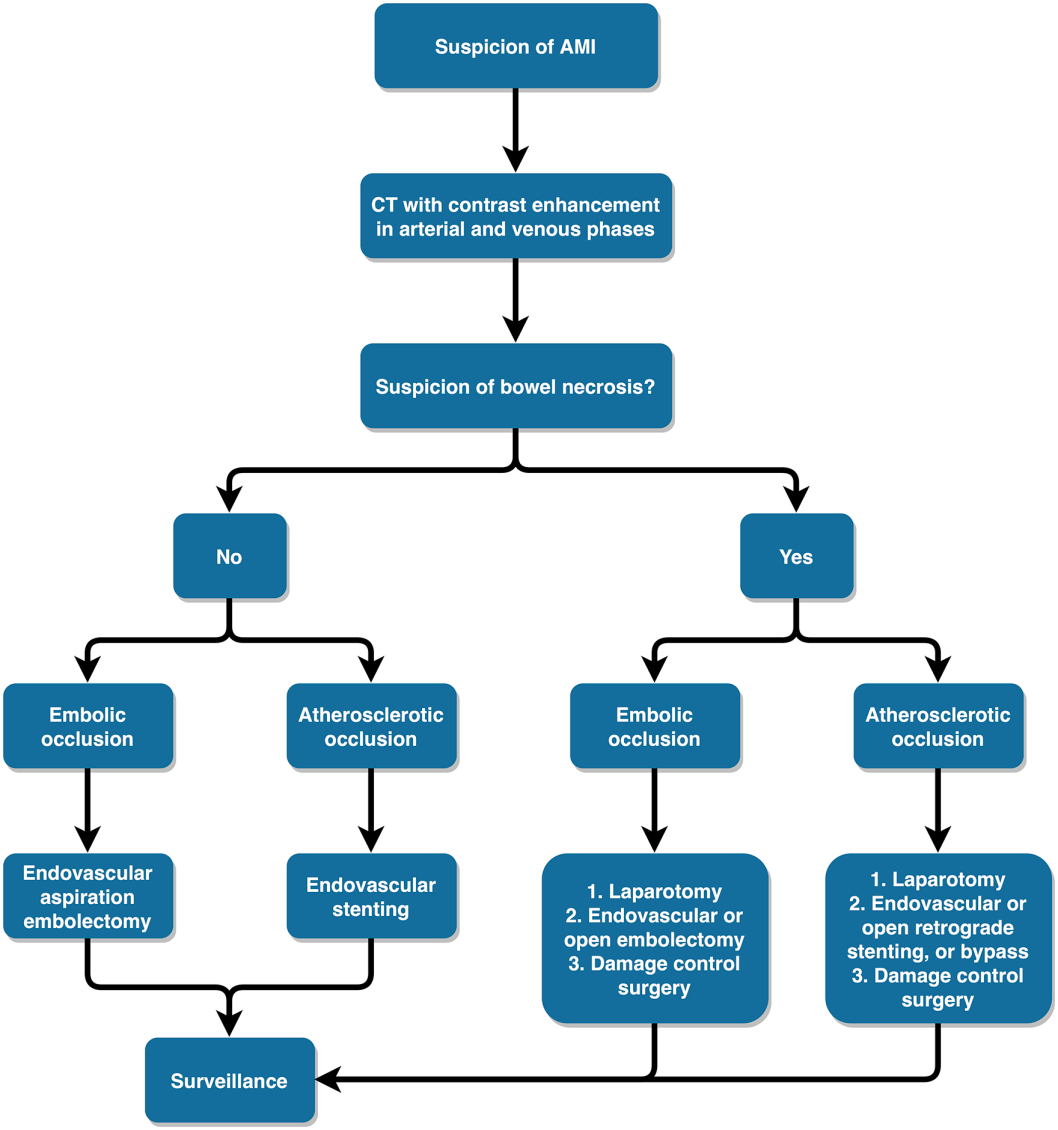
The treatment algorithm for patients with suspicion of acute mesenteric ischemia (AMI) based on computed tomography (CT) and clinical findings.

After the diagnosis of AMI is evident or highly suspected based on CT and clinical findings, the next step is to evaluate whether the patient needs laparotomy to assess bowel viability and damage control or if the patient can undergo an attempt at endovascular revascularization of the SMA without an initial laparotomy (Fig. 6). Diagnosing transmural bowel necrosis and evaluating the need for laparotomy is one of the most crucial decisions the acute care surgeon has to make. A recent meta-analysis identified several clinical features (such as organ failure, systemic inflammatory response syndrome, long duration of symptoms, coronary artery disease, and shock), biochemical markers (such as elevated serum lactate, acidosis, leukocytosis, hemoconcentration, hyperamylasemia), and radiological findings (such as bowel loop dilatation, pneumatosis, arterial or venous mesenteric occlusion, free intraperitoneal fluid) that may suggest increased risk for bowel necrosis (29).

If transmural bowel necrosis is suspected based on clinical signs of peritonitis, septic shock, or CT signs of advanced bowel ischemia, the patient should be rushed to the operating room, preferably to a hybrid room with angio-suite capabilities. In a septic patient, frankly necrotic bowel should be quickly stapled off sparingly with damage control principles. If the patient is stable and there is no bowel leakage, revascularization should be performed before bowel resection because it may be unclear what part of the bowel is still salvageable before restoration of the blood flow (4). In embolic AMI, open surgical embolectomy may be the quickest way to restore blood flow if the laparotomy is already done. In case of atherosclerotic AMI, it is worthwhile to try endovascular stenting and leave surgical bypass as the last resort. If the patient needs laparotomy for bowel assessment, the quickest way to recanalize atherosclerotic occlusion of the SMA may be retrograde open mesenteric stenting (30). The SMA is exposed below the transverse mesocolon and punctured in its main trunk. The proximal SMA lesion is crossed from the retrograde direction with a guidewire. This guidewire is then snared in the aorta using another wire from the femoral artery to establish a stable through-and-through access, and balloon dilatation and stenting of the SMA lesion can then be performed with ease.

In a stable patient with no immediate need for laparotomy, endovascular revascularization can be attempted in a hybrid room or in an angio suite. In case of embolic SMA occlusion, mechanical aspiration thrombectomy is often successful using catheters designed for the purpose. The drawback of this technique is the risk of embolization of the clot particles further into the mesenteric arteries. However, this rarely has any clinical sequela due to the extensive collateral network in the mesentery. If there are significant amounts of residual emboli, a thrombolysis catheter can be left in the SMA for 24 h with close surveillance in the intensive care unit. In cases of atherosclerotic SMA occlusion, endovascular recanalization can be attempted from the femoral, brachial, or radial artery. Stenting is recommended after initial balloon angioplasty of the lesion (4). If the symptoms do not quickly resolve after endovascular revascularization, laparotomy has to be performed without hesitation. In our series of 50 patients who received an initial attempt at endovascular treatment, 60% did not require subsequent laparotomy (2).

After successful revascularization, when bowel resection is required, it should be remembered that the principles of cancer surgery do not apply. There is no need to cut out the mesentery. On the contrary, the mesentery with all the important marginal collaterals should be spared and the resection line should be kept close to the bowel. After bowel resection, the surgeon needs to decide between primary anastomosis and leaving the abdomen open. We do not recommend primary anastomosis if the patient is hemodynamically unstable (requiring vasoactive drugs), the abdomen is seriously contaminated, massive bowel resection or several bowel anastomoses are needed, the abdomen cannot be closed without tension, or the perfusion of the bowel ends are still compromised for any reason. In any of these situations, the stapled bowel ends are left in the abdomen, the abdomen is left open, and a vacuum dressing is applied (31). The abdomen is re-entered usually after 36–48 h (32). If the patient is stable at second look, the bowel anastomoses are performed, and the abdomen is closed. If the patient’s condition is still critical, bowel ends may be converted to double-barrel ostomy; the same thing applies in case of anastomotic leakage.

## Medical Therapy after Initial Treatment of AMI

After revascularization, patients should be started on subcutaneous heparin to prevent further thromboembolic complications. Patients with embolic AMI may be in need of lifelong anticoagulation therapy. Patients with atherosclerotic AMI should be treated with lifelong platelet antiaggregation medication, typically low-dose aspirin, along with modern medical therapy for secondary prevention of the atherosclerotic vascular disease. After SMA stenting, the patient is usually started on clopidogrel for a minimum of 1 month together with the lifelong aspirin to prevent acute stent thrombosis (5). However, there is no scientific data on dual antiplatelet therapy after SMA stenting and the recommendation is based on the data from coronary artery interventions. The SMA is a high-flow vessel and acute stent thrombosis in the SMA is rare (33). In-stent restenosis that may cause reoccurrence of chronic mesenteric ischemia or a new episode of AMI is often caused by intimal hyperplasia, which unfortunately cannot be avoided with clopidogrel. If the patient develops gastrointestinal bleeding or other bleeding issues after stenting of the SMA, the clopidogrel treatment may be stopped.

## Summary

AMI appears to be relatively common in elderly patients. This is important to acknowledge, because clinical suspicion is a major factor in the early diagnosis and in the interpretation of CT findings. The clinical presentation of AMI varies a great deal depending on the etiology and the presentation pattern of the arterial obstruction. The role of laboratory examinations in the early diagnosis of AMI is limited. In arterial AMI, the ideal facility to treat the patient is a hybrid operating room, where the initial treatment is endovascular revascularization, and laparotomy is performed only on demand. However, the surgeon must not hesitate to perform more aggressive solutions if the “endovascular first” option does not seem suitable. In slowly progressing acute-on-chronic mesenteric ischemia, there is often time to transfer the patient to a dedicated vascular unit.
